# The disease spectrum of adult patients at a tertiary care center emergency department in Lebanon

**DOI:** 10.1371/journal.pone.0216740

**Published:** 2019-05-22

**Authors:** Eveline Hitti, Mirabelle Geha, Dima Hadid, Rana Bachir

**Affiliations:** Department of Emergency Medicine, American University of Beirut Medical Center, Beirut, Lebanon; Stanford University, UNITED STATES

## Abstract

**Objective:**

There is an increase in Emergency Department (ED) utilization globally. Understanding what patients present to EDs with is important for resource allocation, training and staffing purposes. There is paucity of data pertaining to ED visit presentations in Lebanon. This study aims at describing the spectrum of diseases among adult patients who present to a tertiary care center in Lebanon, an upper-middle income country (UMIC).

**Methods:**

A retrospective chart review of adult patients (age ≥ 19) presenting to a tertiary care hospital ED during 2010–2011 was completed. Common diagnoses in three categories (all adult visits, treat and release, admitted visits) were assessed. Diagnoses were classified according to the Clinical Classifications Software. Descriptive statistics were presented in tables as frequencies and percentages.

**Results:**

During the study period, 32787 adults presented to the ED with 18.7% resulting in hospital admission. The most common diagnoses in ED patients were injuries and conditions due to external causes, abdominal pain, non-specific chest pain and intestinal infections. In the treat and release group, intestinal infections emerged in the common list for ages 19–44. Coronary atherosclerosis was common in admitted patients aged ≥45 years. Summer was the busiest season, with abdominal pain and intestinal infection being prominent diagnoses during that season.

**Conclusions:**

This study is the first to assess adult ED visits in a Lebanese setting. Our study suggests that patients in our population suffer from the double burden of both communicable and non-communicable disease, with coronary atherosclerosis common in admitted patients (≥ 45 years) and intestinal infections common in treat and release adult patients (19-44years), the latter condition peaking in summer and driving seasonal surges in ED visits.

## Introduction

There is a substantial increase in emergency department (ED) visit rates globally [1]. In the US, ED visits have been steadily rising since the 1990’s reaching a 10-year high for all age groups in 2015 [2]. In Australia, there is a reported 3.5% yearly surge in visits at several training-accredited EDs, while Canadian EDs have witnessed nearly 12 million visits annually, with a latest rise in the utilization rate by 6% in Ontario [1]. This is not different than the European region where multiple countries have observed increasing ED utilization[1]. This rise in ED visits has also been documented in the developing world including Iran, Saudi Arabia and India [1]. While many data on patterns of ED utilization in the developed countries is available, there is little on patterns of ED utilization in developing countries due to the incompleteness of the medical records, lack of national level data and restriction of existing studies to individual institutions [3, 4]. Understanding the types of presentation, acuity, and admission rates is important for resource utilization, health care planning and training purposes.

In Lebanon, Emergency Medicine is a relatively newly established specialty. While there are around 185 hospitals in Lebanon, clear criteria for ED categorization is lacking, leading to wide variability in staffing, training, equipment and medication availability [5]. Only recently was the first Emergency Medicine training program established (2012), leaving most EDs staffed by a mix of general practitioners, residents or surgeons. Most patients arrive to the ED by private transport (self or family), while the minority present through ambulance services. Emergency medical services remain decentralized, dominated by two main agencies, the Civil Defense and the Lebanese Red Cross, and focus on rapid transport to hospitals rather than pre-hospital medical care[5]. Mirroring the nascency of the specialty, there is a paucity of data pertaining to ED visit presentations in Lebanon.

While there are few studies describing the incidence of specific conditions at specific catchment areas, there is no national comprehensive data on the types of overall presentations to the ED. One study looked at the incidence of acute allergic reactions in the ED, and found that even though the incidence was higher than in other studies, the majority were mild presentations[6]. Another study assessed thrombolytic use in acute stroke patients and found that it was higher than expected [7]. A study describing adult and pediatric trauma patients referred to a tertiary care center in Lebanon found that fall from less than 15 feet (38.2%) was the most common mechanism of injury [8]. The lack of comprehensive assessment of types of presentations, however, continues to pose a challenge for resource planning and staff development.

This study aims to describe the spectrum of diseases among the adult population that presents to a tertiary care center in Lebanon, an upper-middle income country (UMIC).

## Methods

### Study setting and design

The study was conducted at the American University of Beirut Medical Center (AUBMC), the largest tertiary care center in Beirut, Lebanon, seeing approximately 48,000 visits annually, of which 72% are adult patients. Patients are triaged to three sections of the ED (High Acuity, Low Acuity and Pediatrics) based on the Emergency Severity Index (ESI) and age. ESI is a 5-level index used in emergency departments to rate patient’s acuity from level 1 (most urgent) to level 5 (least urgent) based on an estimation of resources required [9]. All non-trauma pediatric cases (18 years of age or less) are seen in the pediatric section. Otherwise, patients are triaged to the High Acuity Section or Low Acuity section based on ESI and chief complaint. The ED is staffed by a mix of American Board-certified/eligible Emergency Medicine physicians and non-emergency medicine trained physicians with extensive practice and ED experience.

The study is a retrospective descriptive study of medical record administrative data of all adult cases that presented to the ED in a one-year period. The study was deemed exempt from human subject research by our Institutional Review Board.

### Sample

The study population included adult patients who presented to the ED between May 2010 and April 2011 defined as patients aged 19 years of age or older. This year was chosen as resource constraints in our medical records department have led to significant lags in coding of ED visits and 2011 was the most recently fully coded year for ED visits.

### Measures

The variables studied include: sociodemographic factors including age (19–44 years old, 45–64 years old, 65–84 years old and ≥85 years old), sex (male/female), payment group (insurance companies/self-paying patients/other), and clinical characteristics (ESI and discharge diagnosis).

### Data source

The data for this study was provided by the Medical Records Department at AUBMC. Diagnoses are coded according to the ICD-9-CM, the International Classification of Diseases, Ninth Revision, and clinical Modification. For admitted patients, the principal diagnosis is the condition that was primarily responsible for the admission of the patient to the hospital for care. For treat-and-release ED visits, the principal diagnosis is the first-listed condition shown in the medical record to be primarily responsible for the services provided. There are approximately 14,000 ICD-9-CM codes. To allow for more manageable trend analysis, the ED diagnoses were further classified according to single–level CCS (clinical classification software), which collapses the ICD-9-CM diagnoses into 285 meaningful diagnoses. Thus, all diagnoses used in this study refer to the single-level CCS categories that the ICD-9 coded diagnoses mapped to, rather than the clinical definitions.

### Statistical analysis

The distribution of the basic socio-demographic characteristics (age, sex, and insurance), and clinical & administrative elements (ESI, and time of admission) were presented in a table as frequencies and percentages. Descriptive statistics were summarized by presenting the numbers and percentages for treat-an-release, admitted and all ED visits for adults by age group and by season.

## Results

A total of 33128 adult patients visit records were retrieved out of which 341 visits were excluded because of missing data, leaving a total of 32787 visits for final analysis.

Table 1 presents the demographic characteristics of adult ED visits from 2010-2011.The total number of ED visits for adults was 32,787 during the study period, of which the majority (81.3%) resulted in treat-and-release while 18.7% resulted in hospital admission.

**Table 1:** Demographic characteristics of adult ED visits 2010–2011.

Variables	Treat-and-release adult ED visit	Adult ED visits resulting in hospital admission	All Adult ED visits*
Number of visits (%)	26665 (81.3%)	6122 (18.7%)	32787 (100.0%)
**Age**			
**Mean (SD), years**	41.3 (18.2)	59.9 (19.2)	44.8 (19.8)
**Median (IQR), years**	37.0 (26–54)	63.0 (45–75)	41.0 (27–60)
**N (%)**			
19–44 years	16623 (91.9%)	1466 (8.1%)	18089 (55.2%)
45–64 years	6321 (78.5%)	1735 (21.5%)	8056 (24.6%)
65–84 years	3393 (57.6%)	2500 (42.4%)	5893 (18.0%)
≥ 85 years	328 (43.8%)	421 (56.2%)	749 (2.3%)
**Sex, N (%)**			
Females	13404 (83.1%)	2731 (16.9%)	16135 (49.2%)
Males	13261 (79.6%)	3391 (20.4%)	16652 (50.8%)
Payment Group, N (%)			
Insurance companies	18721 (84.5%)	3433 (15.5%)	22154 (67.6%)
Self-paying patients	7738 (74.8%)	2613 (25.2%)	10351 (31.6%)
Others	197 (72.7%)	74 (27.3%)	271 (0.8%)
**ESI, N (%)**			
Low acuity (ESI 4 and 5)	9268 (96.7%)	318(3.3%)	9586 (29.2%)
Intermediate acuity (ESI 3)	14611 (78.9%)	3907 (21.1%)	18518 (56.5%)
High acuity (ESI 1 and 2)	2777 (59.5%)	1893 (40.5%)	4670 (14.2%)
**Admission time, N (%)**			
08:00–15:59	10564 (39.6%)	2363 (38.6%)	12927 (39.4%)
16:00–23:59	11317 (42.4%)	2983 (48.7%)	14300 (43.6%)
24:00–07:59	4784 (17.9%)	776 (12.7%)	5560 (17.0%)

*Reflects column percentages for all variables except Number of visit.

Visits by patients between the ages of 19 to 44 years old constituted the highest proportion of ED visits (55.2%) and they were also the age group most likely to be discharged from the ED without admission (91.9%). While patients 85 years of age and older accounted for only 2.3% of ED visits, 56.2% of these patients required admission to the hospital. The majority of patients (56.5%) were triaged as intermediate acuity (ESI 3), while around 29.2% were triaged as low acuity (ESI 4 and 5) and 14.2% as high acuity (ESI 1 and 2). Most of the visits occurred in between 16:00 and 23:59 (43.6%) and the majority of the patients had private insurance (67.6%). Females comprised 49.2% of all ED visits. As compared to females, males made up the larger proportion of admitted patients (20.4% vs 16.9%).

Table 2 presents in-ED and in-hospital mortality for all adult ED visits. The in-ED visit mortality was 0.3% while in-hospital mortality of patients admitted to the hospital from the ED was 4.0%. The majority of adult mortalities were male (54.9%). The top common diagnoses for in-ED visit mortality was *cardiac arrest and ventricular fibrillation* (63.9%), of which the majority presented to the ED in cardiac arrest. The second lead cause of in-ED visit mortality was *Coma*,*Stupor*, *Brain Damage* (8.3%), while *septicemia* (13.8%) and *acute cerebrovascular disease* (8.9%) were the top diagnosis for the in-hospital mortality group.

**Table 2:** In-ED and In-hospital mortality.

Variables	In-ED mortality	In-hospital mortality*
**Total Visit**	32787	6122
**Number of in-visit Deaths (%)**	108 (0.3%)	246 (4.0%)
**Age**		
**Mean (SD), years**	66.26 (18.431)	70.59 (16.421)
**Median (IQR)**	71.5 (56–81)	75.0 (61–82)
**Sex, N (%)**		
Females	31 (28.7%)	111 (45.1%)
Males	77 (71.3%)	135 (54.9%)
** Top common diagnoses**		
Septicemia (except in labor)		34 (13.8%)
Acute cerebrovascular disease		22 (8.9%)
Cancer of bronchus; lung		14 (5.7%)
Congestive heart failure; nonhypertensive	3 (2.8%)	14 (5.7%)
Coronary atherosclerosis and other heart disease		11(4.5%)
Cancer of breast		9 (3.7%)
Cardiac arrest and ventricular fibrillation	69 (63.9%)ϯ	
Coma; stupor; and brain damage	9 (8.3%)	
Other lower respiratory disease	5 (4.6%)	
Intracranial Injury	3 (2.8%)	
Headache; including migraine	2 (1.9%)	
Other injuries and conditions due to external causes	2 (1.9%)	

*Hospital mortality for patients admitted from ED.

ϯ 83% (57/69) arrived to the ED in cardiac arrest.

Table 3 presents the top most common conditions for adult ED visits. The most common conditions for adults ED visits were in descending frequencies; other injuries and conditions due to external causes (9.6%), abdominal pain (9.3%) and nonspecific chest pain (4.1%). While the main diagnosis of females presenting to the ED was abdominal pain (10.8%), for males it was other injuries and conditions due to external causes (10.8%).

**Table 3:** Top most common reasons for all ED visits for adults.

	Male		Female		Total	
Most common conditions ^1^	N (16652)	%	N (16135)	%	N (N = 32787)	%
Other injuries and conditions due to external causes	1804	10.8%	1342	8.3%	3146	9.6%
Abdominal pain	1305	7.8%	1746	10.8%	3051	9.3%
Nonspecific chest pain^**2**^	846	5.1%	484	3.0%	1330	4.1%
Intestinal infection^**3**^	571	3.4%	684	4.2%	1255	3.8%
Other lower respiratory disease^**4**^	533	3.2%	563	3.5%	1096	3.3%
Headache; including migraine	418	2.5%	526	3.3%	944	2.9%
Spondylosis; intervertebral disc disorders; other back problems	450	2.7%	491	3.0%	941	2.9%
Coronary atherosclerosis and other heart disease	416	2.5%	134	0.8%	550	1.7%
Other upper respiratory infections^**5**^	383	2.3%	427	2.6%	810	2.5%
Conditions associated with dizziness or vertigo	352	2.1%	454	2.8%	806	2.5%
Fever of unknown origin^**6**^	413	2.5%	370	2.3%	783	2.4%
Cardiac dysrhythmias	362	2.2%	374	2.3%	736	2.2%
Open wounds of extremities	419	2.5%	303	1.9%	722	2.2%
Nausea and vomiting	190	1.1%	304	1.9%	494	1.5%
Other connective tissue disease	340	2.0%	359	2.2%	699	2.1%

^1^Clinical Classifications Software (CCS) categories based on International Classification of Diseases, Ninth Revision, and Clinical Modification (ICD-9-CM) diagnoses.

^2^ The most frequent ICD-9-CM diagnosis within this CCS category was other chest pain (97.3%).

^3^ The most frequent non-mutually exclusive ICD-9-CM diagnoses within this CCS category were diarrhea of presumed infectious origin *(64*.*7%)*, *colitis*, *enteritis*, and *gastroenteritis* of presumed *infectious origin (18*.*5%)*, and *food poisoning unspecified (5*.*9%)*.

^4^ The most frequent non-mutually exclusive ICD-9-CM diagnoses within this CCS category were other respiratory abnormalities (34.0%), cough (31.6%) and shortness of breath (13.5%).

^5^ The most frequent non-mutually exclusive ICD-9-CM diagnoses within this CCS category were *acute* upper respiratory infections of *unspecified* site (56.3%), and acute pharyngitis (34.8%).

^6^ The most frequent non-mutually exclusive ICD-9-CM diagnoses within this CCS category were *pyrexia* of *unknown origin* (95.4%), and *fever unspecified (4*.*5%)*.

Table 4 shows the most common diagnoses for adult ED visits split by age groups. For the youngest group (19–44 years old), the most common ED diagnosis was other injuries and conditions due to external causes (12.0%) followed by conditions related to abdominal pain (11.0%). Patients between the age range 45–64 years old also presented to the ED with similar diagnoses. However, abdominal pain (9.4%) preceded other injuries and conditions due to external causes (7.6%). The latter was also the most common diagnosis in the 65–84 years old age group (5.7%), followed by coronary atherosclerosis and other heart disease (5.1%), and abdominal pain (4.8%). As for the oldest age group (≥85 years old), they were mostly diagnosed with pneumonia (6.5%), followed by coronary atherosclerosis and other heart disease (5.5%) and fracture of the femoral neck (4.7%).

**Table 4:** Most common reasons for all ED visits for adults by age group.

All-listed conditions^1^	Number (percent) of all ED visits for adults by age group, 2010							
	19–44 years N (18089)	Rank	45- 64years N (8056)	Rank	65–84 years N (5893)	Rank	≥85 years N (749)	Rank
Other injuries and conditions due to external causes	2168 (12.0%)	1	610 (7.6%)	2	334 (5.7%)	1	34 (4.5%)	4
Abdominal pain	1985 (11.0%)	2	760 (9.4%)	1	280 (4.8%)	3		
Intestinal infection^**2**^	934 (5.2%)	3						
Other upper respiratory infections^**3**^	691(3.8%)	4						
Headache; including migraine	627 (3.5%)	5						
Nonspecific chest pain^**4**^			497 (6.2%)	3				
Coronary atherosclerosis and other heart disease					301 (5.1%)	2	41 (5.5%)	2
Conditions associated with dizziness or vertigo			276 (3.4%)	4				
Urinary tract infections							33 (4.4%)	5
Essential hypertension					264 (4.5%)	4		
Pneumonia (except that caused by tuberculosis or sexually transmitted disease)							49 (6.5%)	1
Other lower respiratory disease^**5**^			273 (3.4%)	5	227 (3.9%)	5		
Fracture of neck of femur (hip)							35 (4.7%)	3

1 Clinical Classifications Software (CCS) categories based on International Classification of Diseases, Ninth Revision, and Clinical Modification (ICD-9-CM) diagnoses.

^2^ The most frequent non-mutually exclusive ICD-9-CM diagnoses within this CCS category for adults aged 19–44 years were diarrhea of presumed infectious origin (63.4%), and *colitis*, *enteritis*, and *gastroenteritis* of presumed *infectious origin* (18.7%)

^3^ The most frequent non-mutually exclusive ICD-9-CM diagnoses within this CCS category for adults aged 19–44 years were *acute* upper respiratory infections of *unspecified* site (56.3%), and acute pharyngitis (36.0%).

^4^ The most frequent ICD-9-CM diagnosis within this CCS category for adults aged 45–64 years was other chest pain (98.0%).

^5^ The most frequent non-mutually exclusive ICD-9-CM diagnoses within this CCS category were cough and other respiratory abnormalities (45–64 years: 26.7% and 31.1%; 65–84 years: 21.6% and 46.7%).

Table 5 shows the most common diagnoses for treat-and-release ED visits split by age groups. Different results pertaining to the most common diagnoses for Treat-and-release patients were present when examining age groups separately. For all patients between the age groups 19–44, 65–84, and ≥85 years old, the most common ED diagnosis was other injuries and conditions due to external causes (13.0%, 9.6% and 10.1% respectively). However, for the age group 45–65 years old, abdominal pain was the most common ED diagnosis (11.9%). It was also found that for the age groups 19–44 and 65–84 years old, the second most common ED diagnosis was abdominal pain (11.9% and 8.0% respectively). Whereas other lower respiratory disease was the second most common diagnosis in patients 85 years and older (8.5%).

**Table 5:** Top listed conditions for adult treat- and release ED visits by age group, 2010–2011.

All-listed conditions ^1^	Number (percent) of treat-and-release ED visits by age group, 2010							
	19–44 years N (16623)	Rank	45- 64years N (6321)	Rank	65–84 years N (3393)	Rank	≥85 years N (328)	Rank
Other injuries and conditions due to external causes	2158 (13.0%)	1	604 (9.6%)	2	326 (9.6%)	1	33 (10.1%)	1
Abdominal pain^**2**^	1979 (11.9%)	2	755 (11.9%)	1	273 (8.0%)	2	26 (7.9%)	3
Intestinal infection^**3**^	900 (5.4%)	3						
Other upper respiratory infections^**4**^	679 (4.1%)	4						
Headache; including migraine	613 (3.7%)	5						
Nonspecific chest pain^**5**^			455 (7.2%)	3	169 (5.0%)	4	12 (3.7%)	5
Conditions associated with dizziness or vertigo			257 (4.1%)	4				
Essential hypertension					169 (5.0%)	4		
Other lower respiratory disease^**6**^			248 (3.9%)	5	207 (6.1%)	3	28 (8.5%)	2
Genitourinary symptoms and ill-defined conditions					152 (4.5%)	5	18 (5.5%)	4

^1^ Clinical Classifications Software (CCS) categories based on International Classification of Diseases, Ninth Revision, and Clinical Modification (ICD-9-CM) diagnoses.

^2^ The most frequent non-mutually exclusive ICD-9-CM diagnoses within this CCS category were abdominal pain, unspecified site (19–44 years: 33.2%; 45–64 years: 24.8%; 65–84 years: 27.8%; ≥85 years: 38.5%), abdominal pain, epigastric (19–44 years: 23.2%; 45–64 years: 25.3%; 65–84 years: 27.5%; ≥85 years:19.2), and abdominal pain, left lower quadrant (≥85 years: 19.2%).

^3^ The most frequent non-mutually exclusive ICD-9-CM diagnoses within this CCS category for adults aged 19–44 years were diarrhea of presumed infectious origin (64.1%), and *colitis*, *enteritis*, and *gastroenteritis* of presumed *infectious origin* (19.3%).

^4^ The most frequent non-mutually exclusive ICD-9-CM diagnoses within this CCS category for adults aged 19–44 years were *acute* upper respiratory infections of *unspecified* site (57.1%), and acute pharyngitis (35.9%).

^5^ The most frequent ICD-9-CM diagnosis within this CCS category was other chest pain (45–64 years: 98.0%; 65–84 years: 95.3%; ≥85 years: 100%).

^6^ The most frequent non-mutually exclusive ICD-9-CM diagnoses within this CCS category were cough (45–64 years: 29.0%; 65–84 years: 23.2%; ≥85 years: 25.0%), and other respiratory abnormalities (45–64 years: 32.7%; 65–84 years: 50.7%; ≥85 years: 53.6%).

Table 6 shows the most common diagnoses for ED visits admitted to the hospital split by age groups. The most common diagnoses in the 19–44 years old age group were appendicitis and other appendiceal conditions (5.1%), other complications of pregnancy (5.1%) and biliary tract disease (3.3%). Coronary atherosclerosis and other heart disease was the most common diagnosis for hospital admission in patients belonging to the 45–64 years old and 65–84 years old age groups (10.7% and 12.0% respectively) followed by pneumonia for both age groups (3.7% and 4.9% respectively). The latter was the most common diagnosis for hospital admission in patients 85 years and older (11.2%) followed by coronary atherosclerosis and other heart disease (9.5%).

**Table 6:** Top listed conditions for adult ED visits who were admitted to the hospital by age group, 2010–2011.

All-listed conditions ^1^	Number (percent) of ED visits resulting in admission by age group, 2010							
	19–44 years N (1466)	Rank	45- 64years N (1735)	Rank	65–84 years N (2500)	Rank	≥85 years N (421)	Rank
Appendicitis and other appendiceal conditions	75 (5.1%)	1						
Other complications of pregnancy	75 (5.1%)	1						
Biliary tract disease	49 (3.3%)	2	58 (3.3%)	3				
Complications of surgical procedures or medical care	40 (2.7%)	3						
Mood disorders	39 (2.7%)	4						
Calculus of urinary tract	37 (2.5%)	5						
Spondylosis; intervertebral disc disorders; other back problems								
							
Coronary atherosclerosis and other heart disease			185 (10.7%)	1	300 (12.0%)	1	40 (9.5%)	2
Acute myocardial infarction			54 (3.1%)	4				
Nonspecific chest pain^**2**^			42 (2.4%)	5				
Conditions associated with dizziness or vertigo								
							
Urinary tract infections					96 (3.8%)	3	30 (7.1%)	3
Essential hypertension					95 (3.8%)	4		
Pneumonia (except that caused by tuberculosis or sexually transmitted disease			64 (3.7%)	2	123 (4.9%)	2	47 (11.2%)	1
Congestive heart failure; nonhypertensive					96 (3.8%)	3	22 (5.2%)	5
Acute cerebrovascular disease					91(3.6%)	5		
Fracture of neck of femur (hip)							28 (6.7%)	4

^1^Clinical Classifications Software (CCS) categories based on International Classification of Diseases, Ninth Revision, and Clinical Modification (ICD-9-CM) diagnoses.

^2^ The most frequent ICD-9-CM diagnosis within this CCS category for adults aged 45–64 years was other chest pain (97.6%).

Table 7 shows the most common ED diagnoses by season during 2010. In winter, other injuries and conditions due to external causes (9.7%) was the most common diagnosis, with upper respiratory infections and nonspecific chest pain also being prominent during that season (4.5% and 4.4% respectively). Whereas in summer, abdominal pain (10.2%) emerged as the most common diagnosis with intestinal infection (6.6%) rising to the top five diagnoses.

**Table 7:** Top listed conditions for adult ED visits by season, 2010.

All-listed conditions	Number (percent) of ED visits by season, 2010							
	Winter (N = 8027)	Rank	Spring (N = 7937)	Rank	Summer (N = 9042)	Rank	Autumn (N = 7781)	Rank
Other injuries and conditions due to external causes	778 (9.7%)	1	831 (10.5%)	1	772 (8.5%)	2	765 (9.8%)	2
Abdominal pain	623 (7.8%)	2	705 (8.9%)	2	925 (10.2%)	1	798 (10.3%)	1
Other upper respiratory infections	362 (4.5%)	3						
Nonspecific chest pain	350 (4.4%)	4	336 (4.2%)	3	309 (3.4%)	4	335 (4.3%)	3
Other lower respiratory disease	310 (3.9%)	5			260 (2.9%)	5	291 (3.7%)	4
Spondylosis; intervertebral disc disorders; other back problems			246 (3.1%)	5			263 (3.4%)	5
Intestinal infection			247 (3.1%)	4	594 (6.6%)	3		

## Discussion

Our study is the first to look at ED presentations at a tertiary care center in Lebanon, an upper-middle income country. Our findings suggest that, at the level of the ED, the double burden of disease is evident, with both communicable and non-communicable diseases (NCDs) driving ED visits. This co-occurrence of traditional infectious diseases alongside NCDs is a challenge facing primarily low and middle-income countries which continue to struggle with containing preventable communicable diseases while at the same time face the emergence of NCDs driven by rapid urbanization, lifestyle changes and aging populations. In Lebanon, even though the emergence of NCDs and continued prevalence of communicable diseases has been documented, the impact on ED visits has not been described [10, 11].

The demographic shift Lebanon experienced over the last few decades indicates that it is an aging country [12]. The proportion of those aged 65+ in 2016 is estimated at approximately 9.6% of the population, up from 7.7% in 2006 [13, 14]. In our study, adults aged 65 years and older made up 20.3% of our overall ED visits as compared to 17.6% in upper income countries where they make up 14.5% of the population, reflecting proportionally higher ED utilization of this group in our study population [15, 16]. While the19-44 year age group makes up (38.2%) [14] of the Lebanese population, they constituted 55.2% of our ED visits. This age group was found to drive the majority of ED visits in other studies [15]. The proportion of ED visits with admission to the hospital increased as age increased among the following groups: 45–64 years (21.5%), 65–84 years (42.4%), and 85 years and older (56.2%). Similar trends have been reported in other studies, reflecting the increased acute care needs of aging patients [15].

In-ED mortality rate in our study was lower than the reported median for LMIC of 1.8% but higher than the in-ED mortality reported for UIC like the US. This could be related to higher ED lengths of stay for patients in LMIC, where longer stays increase likelihood of in-ED mortality [17]. In addition, regulatory differences related to termination of resuscitation (TOR) in the field could influence in-ED mortality. While some countries follow field TOR guidelines that limit transportation of such patient to EDs, this is not the case in Lebanon as reflected by our data where more than 50% of in-ED mortality is related to cardiac arrest, with the majority of those patients having arrived to the ED in cardiac arrest [18].

Males had a higher overall percentage of ED visits than females (50.8% versus 49.2%). This is not in line with the literature from upper middle-income countries like Central Anatolia where females had a higher overall percentage of ED visits [19]. Moreover, the percentage of ED visits that resulted in discharge is higher among females than males (79.6% vs 83.1%). This is in accordance with literature from the US where the percentage of ED visits resulting in discharge was over 20 percent higher for females than males [15]. Exploring reasons for this, whether related to differences in patient or illness characteristics by gender, or extrinsic factors like differences provider decision making tied to patient gender, is an area for further research.

The disease pathology reported in our study also reflects the double burden trends seen in other developing countries. Although nonspecific chest pain and coronary atherosclerosis and other heart diseases were prominent among 45–64 years old and 65 years old adults respectively, intestinal infections, urinary tract infections and pneumonia were among the most common conditions for ED visits of adults aged 19–44 years old and adults aged 85 years and older respectively. Studies from lower-middle countries like Bangladesh, also reported heart diseases to account for the majority of NCDs [20].

In our study, only 18.7% of patients were admitted to the hospital with 81.3% of visits resulting in treat-and-release. This is relatively similar to data from other upper middle income countries like Turkey (17.6%) [3, 19], and data from upper income countries like the US, where five times as many patients were discharged from the ED as were admitted [15]. The EM model of care that we practice focuses on reducing admission rates, where extensive work ups are performed in the ED to identify life or limb-threatening conditions and only patients who require further management beyond the initial diagnostic and stabilization phase are admitted.

While other injuries and conditions due to external causes and abdominal pain and intestinal infection emerged among treat-and-release adults aged 19–44 years, nonspecific chest pain ranked 3^rd^ and 4^th^ in the 45–64 and 65–84 year old age groups, respectively. This is in line with literature from upper income countries where abdominal pain, sprains and strains emerged within the most common diagnoses among adults aged 19–44 years old and non-specific chest pain among adults aged 45–64 and 65–84 years old. However, intestinal infections were absent from the common diagnoses list of adults aged 19–44 years old in the literature from upper income countries [15]. This is likely reflecting differences in public health standards, food quality and safety practices in different countries. For patients aged 65–84 years and ≥85 years, superficial injury and contusion was absent from the top diagnosis at our ED, whereas it emerged among the most common diagnoses resulting in discharge in upper income countries like the US [15]. One reason for this could be that in Lebanon, it is common culture that caring for the older family members is a family responsibility, with little reliance on nursing care facilities [21].

While appendicitis and appendiceal conditions, pregnancy complications, biliary tract diseases and complications of surgical procedures or medical care mainly emerged among admitted adults aged 19–44 years, cardiovascular diseases emerged as a prominent NCD among admitted adults aged 45 years and older. This is in line with other studies in the Middle East, Africa and Asia [22]. Coronary atherosclerosis prominently emerged as a top diagnosis resulting in hospital admission in patients aged ≥45 and congestive heart failure emerged as a top diagnosis resulting in admission for people 65 years and older. Results of this study are also similar to existing literature from upper income countries like the US [23].

Mood disorders in patients aged 19–44 years admitted to the hospital ranked 4^th^ in our setting, whereas in the US, it was the top most diagnosis resulting in hospital admission accompanied by schizophrenia and other psychotic disorders [15]. The difference could however reflect stigmatization that accompanies mental health in the Middle East as well as lack of third party payer coverage of psychiatric care in Lebanon which could result in fewer patients seeking care for mental illness in our setting [24, 25].

Understanding seasonal variations of ED visits has implications on staffing and resource requirements. A study by Holleman et al. found that EDs in winter months experience higher patient volumes than summer. In our study, however, summer was the busiest season with abdominal pain ranking 1^st^ and intestinal infection 3^rd^. This may be related to food hygiene and storage in a country where power outrages are still common and the climate is subject to high temperatures in summer. Winter was the second busiest season in our study with a high number of other upper and other lower respiratory tract infections. This is consistent with other studies that found a rise in respiratory related ED visits during wintertime [26].

## Limitations

One of the main limitations of our study is that it includes data from only one medical center in Beirut; an urban tertiary care setting that might not reflect national or global trends. It is however the busiest ED in Lebanon seeing the highest loads of patient annually. Another limitation is the paucity of demographic data available for analysis. Finally the retrospective study design depends on the ICD-9-CM coding of our medical records department, which may be subject to human error as well as limitations of what is documented in the chart, and thus the level of accuracy of the ultimate ICD-9-CM diagnosis assigned. Finally, although the use of CCS categories was important for comparative purposes, not all categories intuitively translate into meaningful clinical diagnosis.

## Conclusion

In conclusion, our study reveals that ED visits in Lebanon, a middle-upper income country, are driven by both NCDs and CDs. Coronary atherosclerosis was mostly common among patients ≥45, while intestinal infections emerged in the top most common conditions overall. Intestinal infections along with abdominal pain together accounted for 16.8% of summer ED visits likely contributing the seasonal summer peaks in ED volume in our setting. This study sheds some light on ED utilization patterns in our setting and can be used to match resources and training to the specific needs of this patient population.

## Supporting information

S1 File Dataset of all adult patients presenting to the ED between May 2010 and April 2011. (SAV)

## Abbreviations

ED: emergency department
ICD-9-CM: The International Classification of Diseases, Ninth Revision, Clinical Modification
CCS: *Clinical Classifications* Software
US: United States
UMIC: upper-middle income country
AUBMC: American University of Beirut Medical Center
ESI: Emergency Severity Index
EM: Emergency Medicine
LMIC: lower-middle income country
